# Draft Genome Sequence of *Clostridium straminisolvens* Strain JCM 21531^T^, Isolated from a Cellulose-Degrading Bacterial Community

**DOI:** 10.1128/genomeA.00110-14

**Published:** 2014-02-20

**Authors:** Masahiro Yuki, Kenshiro Oshima, Wataru Suda, Mitsuo Sakamoto, Keiko Kitamura, Toshiya Iida, Masahira Hattori, Moriya Ohkuma

**Affiliations:** a Biomass Research Platform Team, RIKEN Biomass Engeneering Program Cooperation Division, RIKEN Center for Sustainable Resource Science, Tsukuba, Ibaragi, Japan; b Center for Omics and Bioinformatics, Graduate School of Frontier Sciences, the University of Tokyo, Kashiwa, Chiba, Japan; c Japan Collection of Microorganisms/Microbe Division, RIKEN BioResource Center, Tsukuba, Ibaragi, Japan

## Abstract

Here, we report the draft genome sequence of a fibrolytic bacterium, *Clostridium straminisolvens* JCM 21531^T^, isolated from a cellulose-degrading bacterial community. The genome information of this strain will be useful for studies on the degradation enzymes and functional interactions with other members in the community.

## GENOME ANNOUNCEMENT

Lignocellulosic biomass, which is a mixture of cellulose, hemicellulose, and lignin, is the most abundant biopolymer in the Earth. In nature, lignocellulosic biomass is degraded by a set of synergistically acting enzymes of various microorganisms. Strain CSK1^T^ (deposited as IAM 15070 and now available from Japan Collection of Microorganisms as JCM 21531^T^) was isolated from a cellulose-degrading bacterial community and described as the type strain of a novel species, *Clostridium straminisolvens* (1, 2). The 16S rRNA gene sequence analysis indicated that *C. straminisolvens* is related to anaerobic cellulolytic bacteria *Clostridium thermocellum* and *Clostridium aldrichii*. *C. straminisolvens* JCM 21531^T^ grows optimally at 50 to 55°C and shows aerotolerance for growth and an ability to ferment cellulose and cellobiose (1). The cellulose-degrading efficiency in pure culture of *C. straminisolvens* JCM 21531^T^ is remarkably lower than that in coculture with aerobic noncellulolytic bacteria, suggesting their synergistic relationships (3, 4).

The genome of *C. straminisolvens* JCM 21531^T^ was sequenced using the Ion Torrent PGM system. The 367,174 sequence reads were assembled using Newbler version 2.8 (Roche) into 195 contigs, with an N_50_ length of 48,174 bp. This assembly resulted in the draft genome sequence of 3,907,117 bp, with 18.7× redundancy and a G+C content of 38.3%. A total of 4,383 protein-coding genes and 53 RNA-coding sequences were identified using the RAST server (5) and with the manual inspections detailed below.

RAST annotations and the following CAZy database analyses (6) revealed that *C. straminisolvens* JCM 21531^T^ has various genes encoding endoglucanases classified in the glycoside hydrolase 5 (GH5), GH8, GH9, GH48, GH74, and GH124 families, genes encoding GH5 and GH9 of cellobiohydrolases, which degrade crystalline cellulose, and genes encoding β-glucosidases of GH1 and GH3. In addition, *C. straminisolvens* JCM 21531^T^ also has several genes encoding xylanases of GH10. The presence of genes encoding nitrogenase and enzymes for the reductive acetyl-coenzyme A (CoA) pathway indicated the potentials of this strain of a diazotrophic and homoacetogenic nature, respectively. Detailed analyses of the genome of this strain, including comparisons with published genome sequences of *C. thermocellum* strains (7–10), will facilitate studies on the nature of cellulose degradation of *C. straminisolvens* JCM 21531^T^ and its synergistic relationships with other bacteria in the cellulose-degrading community.

### Nucleotide sequence accession numbers.

The genome sequence of *C. straminisolvens* JCM 21531^T^ has been deposited in the DDBJ/EMBL/GenBank database under the accession no. BAVR01000001 to BAVR01000195.

## References

[1] Kato S Haruta S Cui ZJ Ishii M Yokota A Igarashi Y 2004 *Clostridium straminisolvens* sp. nov., a moderately thermophilic, aerotolerant and cellulolytic bacterium isolated from a cellulose-degrading bacterial community. Int. J. Syst. Evol. Microbiol. 54:2043–2047. 10.1099/ijs.0.63148-0 15545431

[2] Haruta S Cui Z Huang Z Li M Ishii M Igarashi Y 2002 Construction of a stable microbial community with high cellulose-degradation ability. Appl. Microbiol. Biotechnol. 59:529–534. 10.1007/s00253-002-1026-4 12172621

[3] Kato S Haruta S Cui ZJ Ishii M Igarashi Y 2004 Effective cellulose degradation by a mixed-culture system composed of a cellulolytic *Clostridium* and aerobic non-cellulolytic bacteria. FEMS Microbiol. Ecol. 51:133–142. 10.1016/j.femsec.2004.07.015 16329862

[4] Kato S Haruta S Cui ZJ Ishii M Igarashi Y 2005 Stable coexistence of five bacterial strains as a cellulose-degrading community. Appl. Environ. Microbiol. 71:7099–7106. 10.1128/AEM.71.11.7099-7106.2005 16269746 PMC1287685

[5] Aziz RK Bartels D Best AA DeJongh M Disz T Edwards RA Formsma K Gerdes S Glass EM Kubal M Meyer F Olsen GJ Olson R Osterman AL Overbeek RA McNeil LK Paarmann D Paczian T Parrello B Pusch GD Reich C Stevens R Vassieva O Vonstein V Wilke A Zagnitko O 2008 The RAST server: Rapid Annotations using Subsystems Technology. BMC Genomics 9:75. 10.1186/1471-2164-9-75 18261238 PMC2265698

[6] Cantarel BL Coutinho PM Rancurel C Bernard T Lombard V Henrissat B 2009 The carbohydrate-active enzymes database (CAZy): an expert resource for glycogenomics. Nucleic Acids Res. 37:D233–D238. 10.1093/nar/gkn663 18838391 PMC2686590

[7] Hemme CL Mouttaki H Lee YJ Zhang G Goodwin L Lucas S Copeland A Lapidus A Glavina del Rio T Tice H Saunders E Brettin T Detter JC Han CS Pitluck S Land ML Hauser LJ Kyrpides N Mikhailova N He Z Wu L Van Nostrand JD Henrissat B He Q Lawson PA Tanner RS Lynd LR Wiegel J Fields MW Arkin AP Schadt CW Stevenson BS McInerney MJ Yang Y Dong H Xing D Ren N Wang A Huhnke RL Mielenz JR Ding SY Himmel ME Taghavi S van der Lelie D Rubin EM Zhou J 2010 Sequencing of multiple clostridial genomes related to biomass conversion and biofuel production. J. Bacteriol. 192:6494–6496. 10.1128/JB.01064-10 20889752 PMC3008519

[8] Feinberg L Foden J Barrett T Davenport KW Bruce D Detter C Tapia R Han C Lapidus A Lucas S Cheng JF Pitluck S Woyke T Ivanova N Mikhailova N Land M Hauser L Argyros DA Goodwin L Hogsett D Caiazza N 2011 Complete genome sequence of the cellulolytic thermophile *Clostridium thermocellum* DSM1313. J. Bacteriol. 193:2906–2907. 10.1128/JB.00322-11 21460082 PMC3133140

[9] Brown SD Lamed R Morag E Borovok I Shoham Y Klingeman DM Johnson CM Yang Z Land ML Utturkar SM Keller M Bayer EA 2012 Draft genome sequences for *Clostridium thermocellum* wild-type strain YS and derived cellulose adhesion-defective mutant strain AD 2. J. Bacteriol. 194:3290–3291. 10.1128/JB.00473-12 22628515 PMC3370843

[10] Koeck DE Wibberg D Koellmeier T Blom J Jaenicke S Winkler A Albersmeier A Zverlov VV Pühler A Schwarz WH Schlüter A 2013 Draft genome sequence of the cellulolytic *Clostridium thermocellum* wild-type strain BC1 playing a role in cellulosic biomass degradation. J. Biotechnol. 168:62–63. 10.1016/j.jbiotec.2013.08.011 23968723

